# Nanostructured CuO Thin-Film-Based Conductometric Sensors for Real-Time Tracking of Sweat Loss

**DOI:** 10.1021/acsomega.3c02232

**Published:** 2023-05-23

**Authors:** Raşit Aydın, Abdullah Akkaya, Osman Kahveci, Bünyamin Şahin

**Affiliations:** † Department of Physics, Faculty of Sciences, Selcuk University, Konya 42130, Turkey; ‡ Mucur Technical Vocational Schools, Tech. Prog. Department, Kırşehir Ahi Evran University, Kırşehir 40100, Turkey; § Department of Physics, Faculty of Sciences, Erciyes University, Kayseri 38039, Turkey; ∥ Department of Basic Sciences, Faculty of Engineering, Necmettin Erbakan University, Konya 42090, Turkey

## Abstract

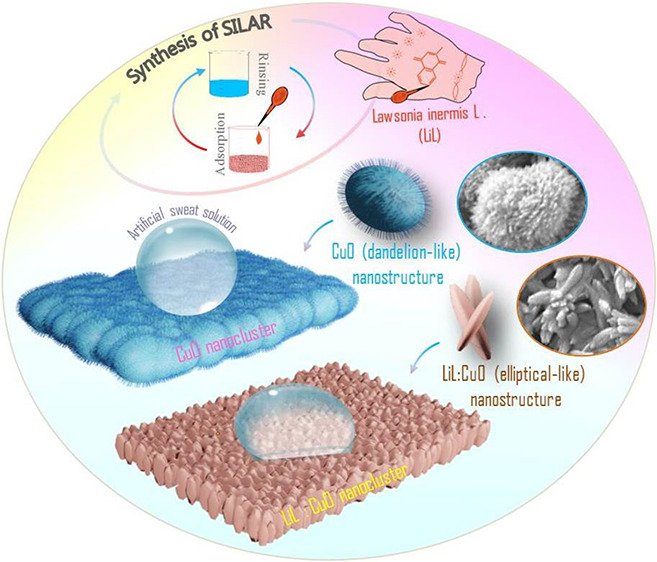

Enhanced sweat sensors lead to real-time, sustained, noninvasive tracking of sweat loss, ensure insight into individual health conditions at the molecular level, and have obtained prominent interest for their hopeful implementations in customized health tracking. Metal-oxide-based nanostructured electrochemical amperometric sensing materials are the best selection for continuous sweat monitoring devices owing to their high stability, high-sensing capacity, cost-effectiveness, miniaturization, and wide applicability. In this research, CuO thin films have been fabricated by successive ionic layer adsorption and reaction technique (SILAR) with and without the addition of *Lawsonia inermis* L. (Henna, (LiL)) leaf extract (C_10_H_6_O_3_, 2-hydroxy-1,4-naphthoquinone) with a high-sensitive and rapid response for sweat solution. Despite the pristine film being responsive to the 65.50 mM sweat solution (*S* = 2.66), the response characteristic improves to 3.95 for the 1.0% LiL-implemented CuO film. Unmodified, 1.0% LiL and 3.0% LiL-substituted thin-film materials assure considerable linearity with linear regression ranges, *R*^2^, of 0.989, 0.997, and 0.998, respectively. It is noteworthy here that this research aims to determine an enhanced system that could potentially be implemented in real-life sweat-tracking administrations. Real-time sweat loss tracking capabilities of CuO samples was found to be promising. Derived from these outcomes, we concluded that the fabricated nanostructured CuO-based sensing system is a useful application for the continuous observation of sweat loss as a biological argument and compatibility with other microelectronic technologies.

## 1 Introduction

Recent progress in material science, structures, and design has led to the advancement of thin, reusable electronic materials that can be conformably usable for monitoring human activity. In particular, electrochemical sensors are used to detect biomarkers used in the diagnosis of metabolic diseases due to their real-time analysis, cheapness, and simplicity. To improve the efficiency of these sensors, carbon nanomaterials such as graphene, carbon quantum dots, and carbon nanotubes are widely used as promising materials. Carbon-based nanomaterials are also used in sensor technology to detect volatile organic compounds such as halogenated hydrocarbons and aromatic hydrocarbons. Graphene and its hybrid derivatives combined with polymers and liquid crystals enable the formation of hybrid nanocomposites with unique properties. Graphene hybrid composites are involved in the production of new interchangeable devices used in applications such as photodetectors and energy storage.^1−4^ Additionally, the continuous monitoring of biological samples underlies much of the health problems and their contemporary medicine.^5,6^

Sweat monitoring can serve a distinctive way in the analysis of biological fluids, ensuring a noninvasive and less complex solution compared to blood analysis. Sweating is the essential procedure to arrange organism temperature; however, it also leads to the loss of water and major electrolytes.^7,8^ Heavy sweating is the main justification for dehydration during exercise activity, particularly in warm or hot conditions.^9,10^

As a biological fluid, sweat is readily obtained from the skin surface of the living body. The balance of electrolytes in the sweat is of vital importance and sweat largely comprises electrolytes such as Na^+^, Cl^–^, and K^+^ and metabolites such as lactate and glucose. The maintenance of sufficient fluid, electrolyte, and metabolite balance is crucial to the overall physical and healthy endurance of an individual.^11,12^ Consequently, real-time observing sweat loss as a biological argument can be used as an appliance to follow the overall physical and health endurance of humans as long as performance.^12^

In recent decades, numerous scientists have been attitude to cultivate healthcare devices for tracking the sweat loss of humans. Among all performed monitoring materials, metal-oxide-based nanostructured semiconductors (MOSs) were preferred by researchers owing to their superiorities such as easy fabrication, acceptable bandgap, long-term stability, fast response, and high sensitivity.^6,13^

Among various MOSs, cupric oxide (CuO) is a monoclinic p-type semiconductor material with a narrow band gap of 1.2 eV. Additionally, CuO is an abundant, inexpensive, electrochemically stable, nontoxic, and easily prepared material. CuO has inspired industry and scientists because of these unique properties compared to existing metal oxides.^14−16^ Films made from CuO are used in a wide variety of technological fields, including superconductors, lithium-ion batteries, diodes, photodetectors, gas sensors, catalysis, biosensors, and solar cell applications. In addition, there are electrochemical applications in the literature where copper is used as an electrocatalyst for nitrite-to-ammonium conversion and nitrogen-to-ammonium conversion.^17−19^ CuO films are especially preferred in sensor applications due to their large surface areas. Larger surface areas in sensors mean a greater probability of adsorption resulting in a better response.^20−23^

When the literature is examined, it is seen that different methods such as chemical bath deposition, spray pyrolysis, evaporation, chemical precipitation method, and successive ionic layer adsorption and reaction (SILAR) are used to synthesize CuO films. SILAR is an easy-to-use, economical, and highly efficient technique in which the thickness and homogeneity of CuO films can be controlled.^24−28^

To examine the physical properties of CuO films, agents such as cetyltrimethylammonium bromide and polyethylene glycol are generally used in synthesis solutions.^29,30^*Lawsonia inermis* L. (Henna, (LiL)) was used as a capping and reducing agent in this study to improve the structural and morphological properties and sweat detection response of the produced CuO films.

Henna is a bio-based reducing and concealing agent that has long been used in medicine and cosmetics in many parts of the world. Henna is commonly known as lawsone (C_10_H_6_O_3_, 2-hydroxy-1,4-naphthoquinone (HNQ, hennotannic acid)), which is a red-orange pigment responsible for its color. Henna has many important advantages such as being healthy, being compatible with nature, exhibiting excellent antimicrobial properties, UV protective properties, and having little chemical reactivity.^31−33^

Because of the hydroxyl group, henna can be used as great artificial sweat solution absorption equipment.^34^ In this research, we used LiL-substituted nanostructured CuO thin films for real-time sweat loss detection as a first step toward the development of a sweat loss tracking material and investigated its main physical characteristics.

## 2 Results and Discussion

**Figure 1:**
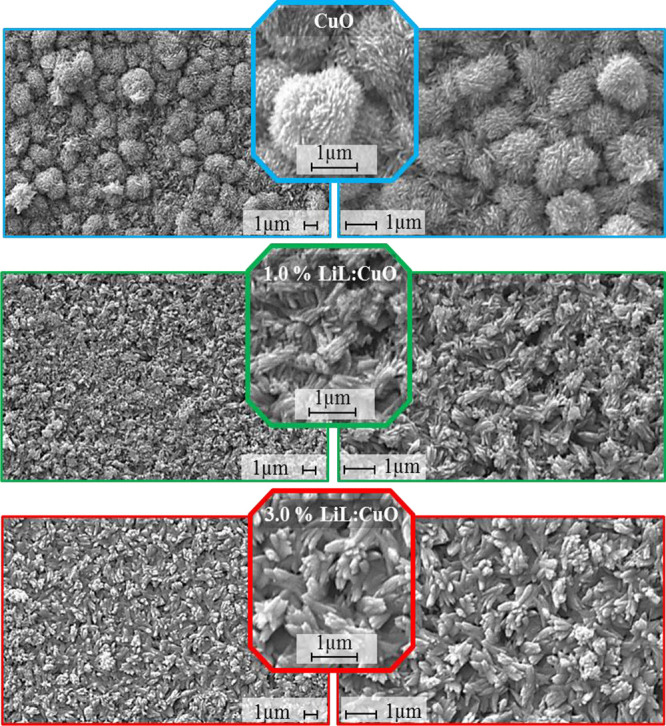
FESEM images of CuO thin films synthesized with various *Lawsonia inermis* leaf extract concentrations at different magnifications. Dandelion-like nanostructures transform into elliptical-like nanostructures with the LiL addition.

FESEM images for the morphological analysis of CuO and LiL-modified LiL:CuO nanostructures are given in Figure 1. 1 μm scale lines are also embedded in all figures. FESEM micrographs were analyzed to examine the effect of different LiL concentrations on CuO nanoclusters formation. Dandelion-like nanostructures are observed in the unmodified CuO sample, LiL:CuO structures obtained with the LiL additive appear to transform into elliptical-like structures (observable in an octagonal shape in magnified images). Both dandelion-like^35,36^ and elliptical-like^37^ structures are nanostructures reported in CuO studies. Additionally, while the surface roughness of nanostructures in the CuO sample was quite high with the presence of hairy tips, the surface roughness decreased in 1.0% LiL:CuO where the concentration ratio in the growth bath was 1.0%, and it was observed that the roughness in the 3.0% LiL:CuO sample, where the concentration ratio in the growth bath was 3.0%, decreased even more. The decrease in the surface roughness of the polycrystalline nanostructure observed in all samples with the addition of LiL is also in agreement with the AFM results. The reduction of the surface capillary with the morphological change in the films with the addition of LiL probably plays a role in strengthening the cohesion effect of the drop of solution on the sample. It has been reported that the morphological change in the films affects the interaction with the solution drop on the device surface.^38^ In all modified and unmodified samples, it is seen that the nanoclusters cover the whole surface properly and there is no stacking fault. It is obvious from the results that the LiL additive has a significant effect on the morphological structure of CuO. From the FESEM images, it was concluded that doping LiL to CuO reduces the rough hairy structure of the nanostructure, resulting in the formation of a smoother surface, and the nanostructure turns into an elliptical form.

**Figure 2:**
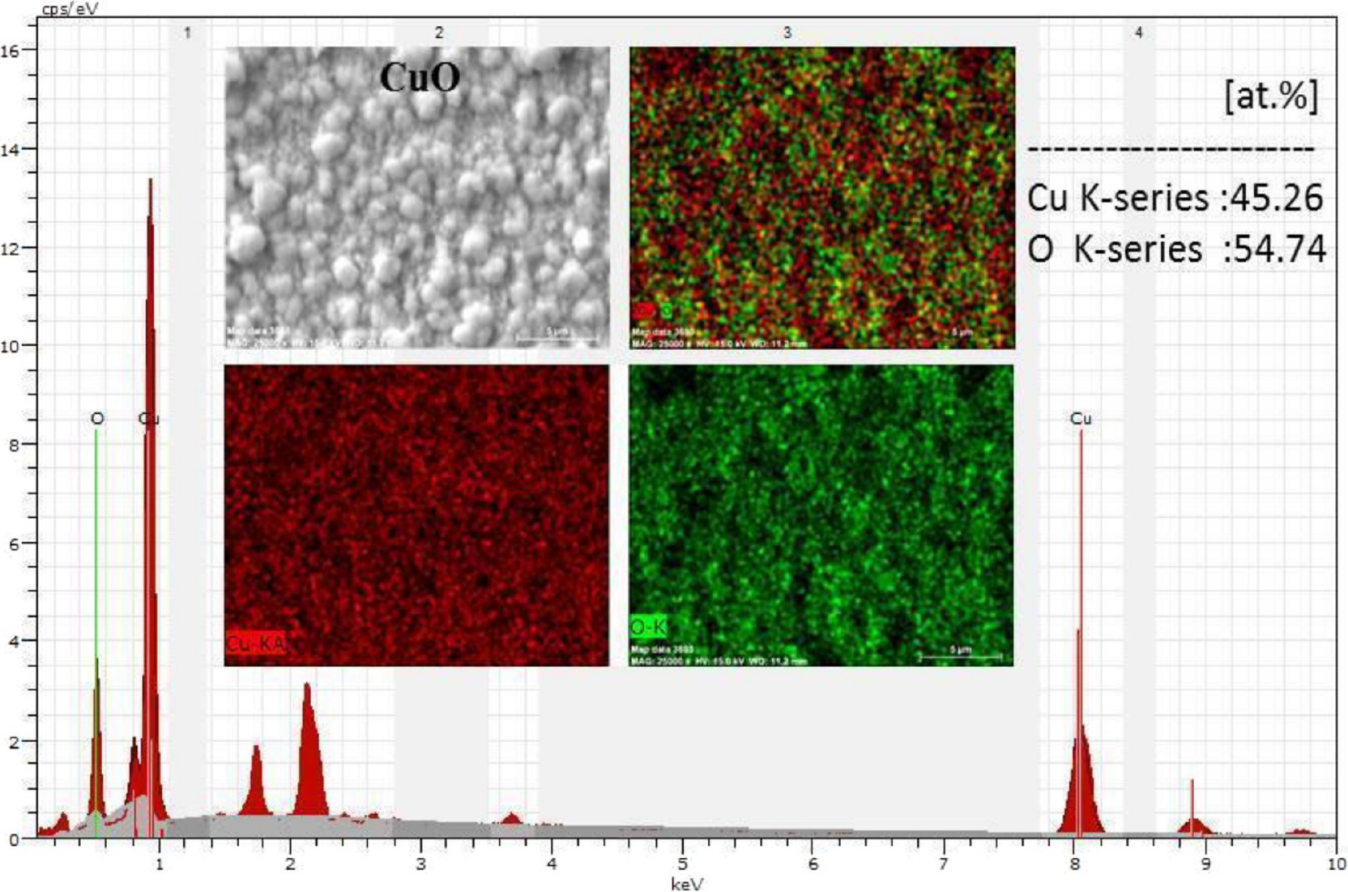
EDX peaks, EDX-mapping images, and elemental analysis result of CuO thin film.

The EDX peak graph and mapping results of the CuO film and embedded elemental analysis result are given in Figure 2. It is seen that the atomic ratios of Cu and O elements are 45.26 and 54.74%, respectively. It has been observed that the measured atomic ratios are also compatible with the literature.^39^ The homogeneous distribution of the film and the presence of the elements were confirmed by EDX results and mapping images.

**Figure 3:**
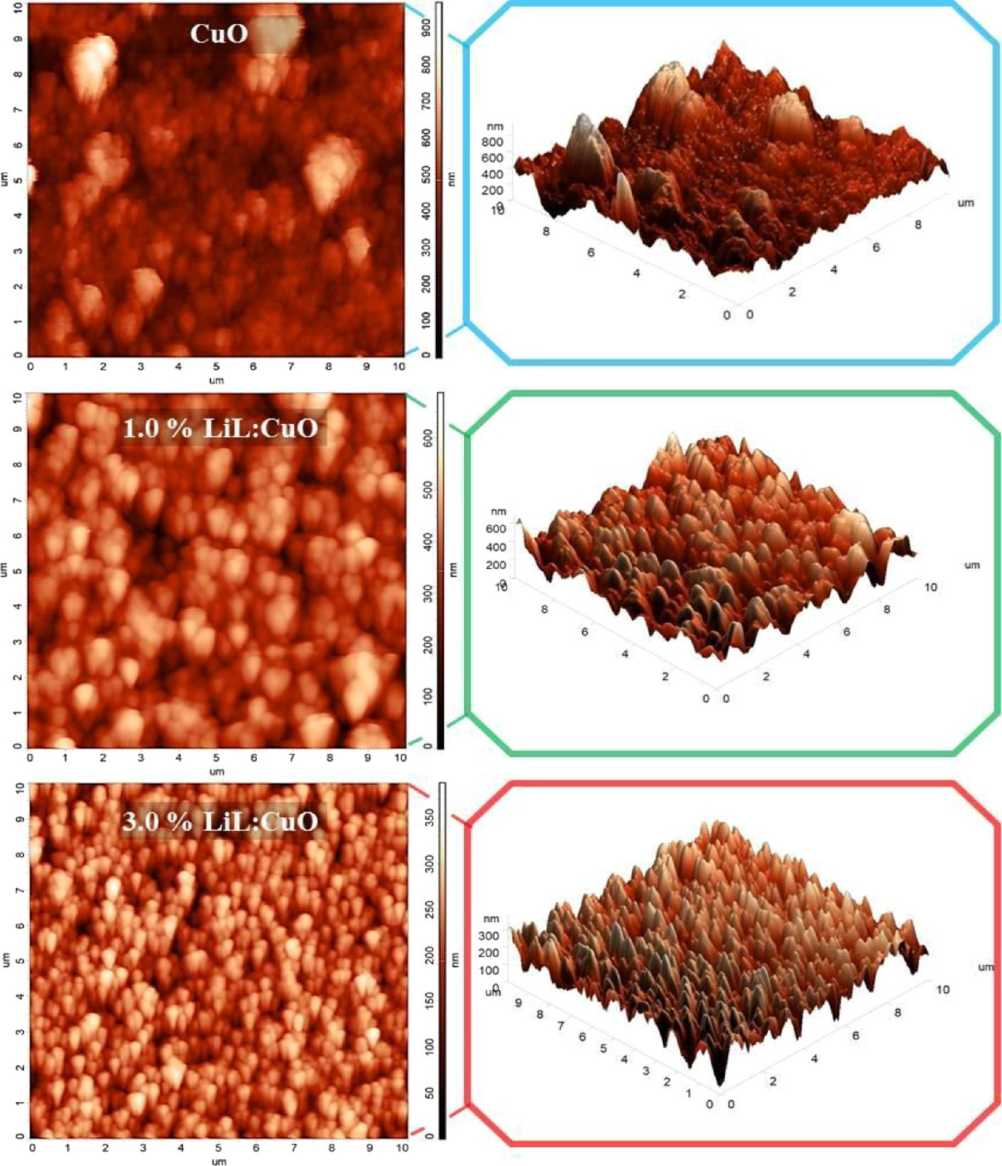
2D and 3D AFM topography images (10 × 10 μm^2^ scan area) of CuO thin films synthesized with various *Lawsonia inermis* leaf extract concentrations. Surface roughness decreased with the increasing LiL concentration.

The atomic force microscopy (AFM) method was used to determine the surface properties of unmodified and 1.0 and 3.0% *L. inermis* added CuO films. This method could reveal a film’s nature and functional performance of films. Figure 3 shows the 2D and 3D AFM topography images (10 × 10 μm^2^ scan area) of films. The surface protrusions in the AFM 3D images decrease as the LiL doping ratio increases. Similar results seem to be consistent with the FE-SEM images. At the same time, the small-width hairy tips in the nondoping CuO structure also refer to the dandelion-like structure. Since this situation has to be examined with a contrast indicator in 2D images, the presence of hairy tips cannot be observed exactly, but in doped structures, it can be easily observed in light color, probably because the ends of the elliptical-like structures are wider.

Micrographs reveal the tightly packed and granular nature of LiL-modified CuO nanostructures. As can be seen in AFM micrographs, unmodified CuO films have a rough surface and the root mean square (RMS) value was 124.305 nm (Table 1). However, this value was significantly reduced to 90.7609 and 60.9433 nm by adding 1.0 and 3.0% *L. inermis* to CuO, respectively. Surface structure parameters Sz (ten-point height), Sa (average surface roughness), Sq (root mean square), and structural entropy are given in Table 1 and all parameters decreased with the increasing LiL ratio. These results were consistent with the FESEM results. Crystallite sizes from XRD results are relatively constant for all samples, and the different types of film growth mechanisms that exist may also possibly explain the decrease in RMS values. LiL-modified growth solution causes less sharp grain edges and changes the dandelion-like nanostructures to elliptical-like structures. Additionally, the thickness of films reduced with the LiL concentration, although there is no change in the SILAR cycles (Table 1). LiL content used as a capping and reducing agent in CuO films may cause this.

**Table 1:** Main Surface Roughness Values and Thickness of Manufactured CuO Films Synthesized without and with *Lawsonia inermis* Leaf Extract

LiL concentration (%)	Sz (nm)	Sa (nm)	RMS (nm)	entropy	film thickness (μm)
0	485.034	90.8587	124.305	12.1373	0.96
1.0	344.489	72.1433	90.7609	11.8789	0.67
3.0	263.971	48.1894	60.9433	11.3196	0.59

**Figure 4:**
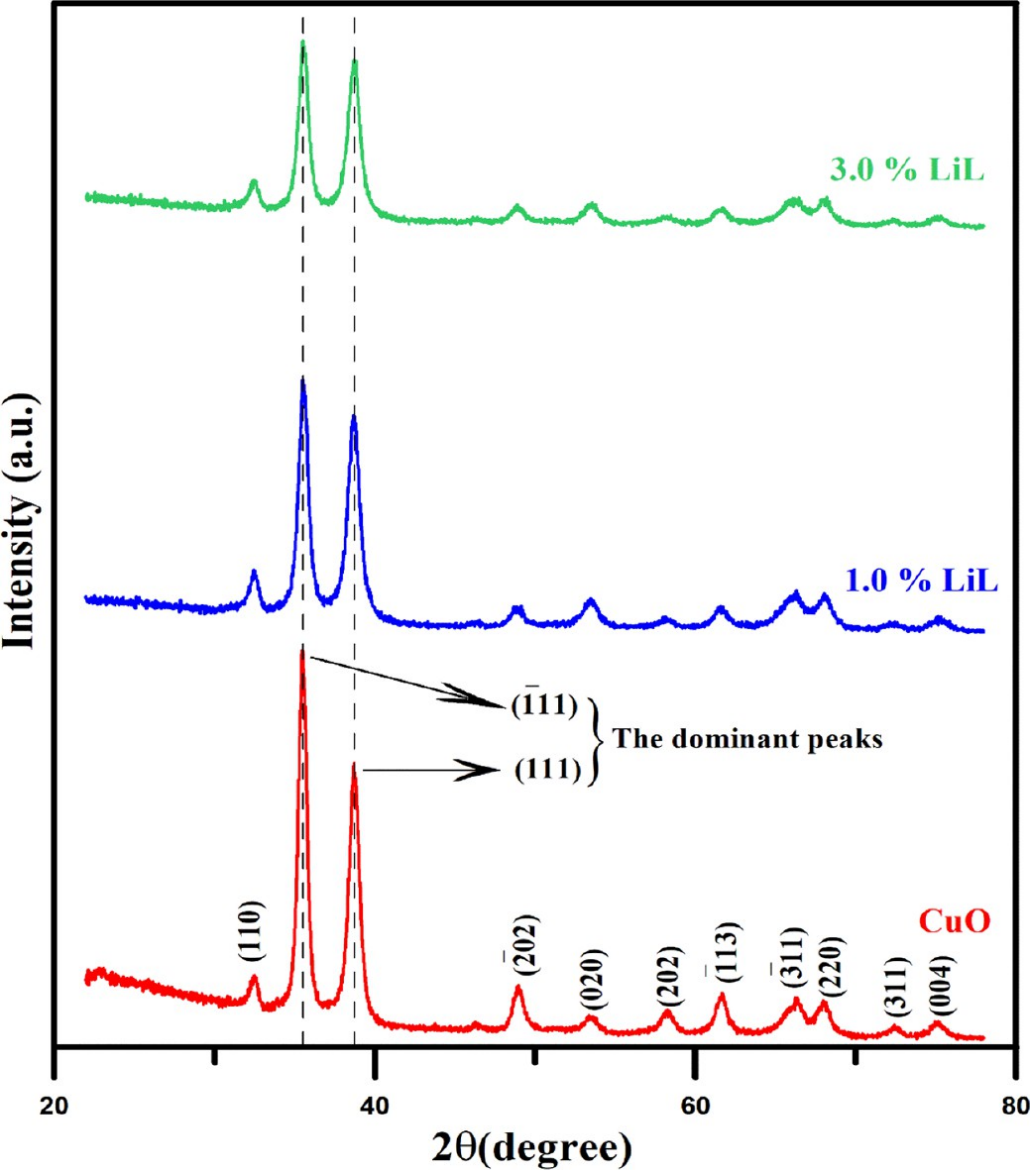
Typical XRD patterns of the CuO samples synthesized with various *Lawsonia inermis* leaf extract concentrations. The peak intensities of the obtained CuO films decrease with increasing LiL percentage.

The effect of LiL content on the crystal structures of the synthesized CuO films was investigated by XRD analysis. The XRD models of CuO films obtained by adding certain percentages of LiL to the reaction solution are shown in Figure 4. It can be seen from Figure 4 that there are almost no diffraction peaks for the CuO films produced, except for the CuO characteristic peaks. The dominant lines at 2θ = 35.50° and 38.66° correspond to  and (111) planes, respectively, and the weak lines at 2θ = 32.45°, 48.87°, 53.37°, 58.26°, 61.54°, 66.25°, 67.88°, 72.48°, and 75.06° correspond to (110), , (020), (202), , , (220), (311), and (004) planes, respectively (JCPDS Card No # 48-11548).^40^ As seen from the XRD pattern, all CuO films produced with and without LiL have a polycrystalline structure. The heights of the peaks of the dense  and (111) planes of the CuO films are presented in Table 2. It is clear from both Figure 4 and Table 2 that the peak heights decrease with increasing LiL content (from 0.0 to 3.0%) in the reaction bath. The decrease in  and (111) peak intensities with decreasing LiL concentration can be attributed to the decrease in the thickness of the CuO films.^41,42^

**Table 2:** Relative Peak Intensity and Crystallite Size Values of CuO Films as a Function of *Lawsonia inermis* Leaf Extract Percentage in the Synthesis Solutions of the SILAR Procedure

LiL concentration (%)	relative peak intensity		FWHM (radian)		crystallite size (nm)
	(1̅11)	(111)	(1̅11)	(111)	
0	1995	1354	0.627	0.800	12.44
1.0	1347	1162	0.697	0.898	11.14
3.0	892	815	0.738	0.938	10.59

The mean crystallite size (*D*) values of planes  and (111) were calculated with the help of Scherrer’s relation by using the values obtained from XRD results such as λ [the wavelength of the CuKα radiation, (1.5406 Å)], β [full width at half maximum (FWHM)], and θ (Bragg diffraction angle).^43^
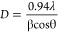
1

It is seen that the calculated *D* values of CuO films decreased from 12.44 to 10.59 nm with increasing LiL concentration in the growth solution (Table 2). As the LiL content increases, the decrease in *D* values may be due to the increase of FWHMs (as seen in Table 2); in other words, the broadening of the dominant XRD peak shapes. Also, this decrease may be due to the increase in crystal lattice distortions after nucleation during the crystallization process with increasing LiL concentration in the solution.^44−46^

**Figure 5:**
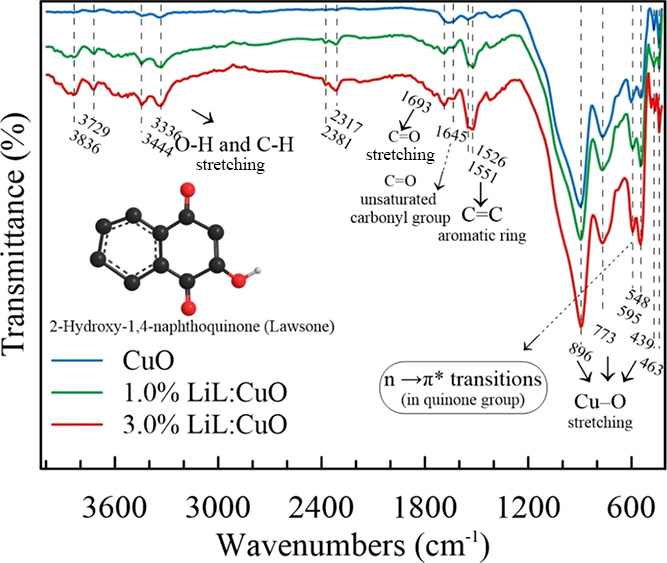
FT-IR absorption spectra of CuO samples synthesized with various *Lawsonia inermis* leaf extract concentrations. Inset shows the lawsone molecule.

Figure 5 shows the Fourier Transform Infrared (FT-IR) spectra of pure CuO and LiL-modified CuO films. Previous studies have shown that the henna extract contains many phenolic compounds (flavonoids, naphthoquinones, quinoids, etc.).^47−49^ However, the two main components of henna are lawsone and gallic acid and the FT-IR spectra mostly contain the peaks of these two components besides the CuO.^31,49,50^ Lawsone molecule (2-hydroxy-1,4-naphthoquinone, C_10_H_6_O_3_), known as hennotannic acid, is reddish-orange dye responsible for the natural color of henna and contains *p*-benzoquinone unit, benzene unit, and phenolic group (see inset in Figure 5).

The IR spectra of CuO revealed strong and sharp absorption bands at the fingerprint region below 1000 cm^–1^. Sharp absorption peaks between 439 and 773 cm^–1^ and broadened and strong peaks appearing at 896 cm^–1^ belong to the typical stretching mode of Cu–O.^27,51−54^ The shifted peak at 595 cm^–1^ can be assigned to the characteristic *n* → π* transitions of quinones coordinated to metals or metal-to-ligand charge-transfer transitions.^55,56^ A few vibration modes were determined in the rest of the FT-IR spectrum and they can be attributed to the organic contamination and residual precursors.^52^ FT-IR spectrum of 1.0 and 3.0% *L. inermis* added CuO films revealed that merged strong absorption bands around 1526 and 1551 cm^–1^ were typical stretching vibrations of C–C bonds in the aromatic ring and indicating the existence of the aromatic group. Two absorption bands at 1693 and 1645 cm^–1^ correspond to the C=O groups in the lawsone molecule. The first absorption peak was shifted (≈17 cm^–1^) to higher wavelengths and belongs to unsaturated C=O and, the second peak belongs to chelated with hydroxyl and/or Cu atoms.^57^ Also, peaks at higher wavelengths 3444 and 3336 cm^–1^ phenolic O–H and C–H stretching modes, respectively.^58^ These bands are feeble, indicating deprotonation and coordination of the hydroxyl oxygen atom.^47,56,59−63^ This results from the signature peaks of *L. inermis* and lawsone, as reported in the articles.^34,60,62,64^

Generally, minor shifts and loss in vibrational frequencies of such a molecule are attributed to their polymeric nature and intra and intermolecular hydrogen bonding tendency with neighboring molecules.^61^ However, if the structure has electronegative atoms, as in lawsone, this bonding becomes more complex and coordination occurs due to the charge delocalization involving the anchoring of the carboxylic group.^47,59−63^ Therefore, this illustrates more stability of the metal center after the coordination of the lawsone ligand. The stability of metals against the corrosion is increased by the donated electron of the phenol group of lawsone and usually revives to explain the corrosion-resistant nature of some lawsone or henna coatings.^49,63−66^ Similarly, the donor ability of hydroxynaphthoquinones is also used to explain an electron transfer mediator in biochemical fuel cells or other neighbor atoms/molecules.^67,68^

**Figure 6:**
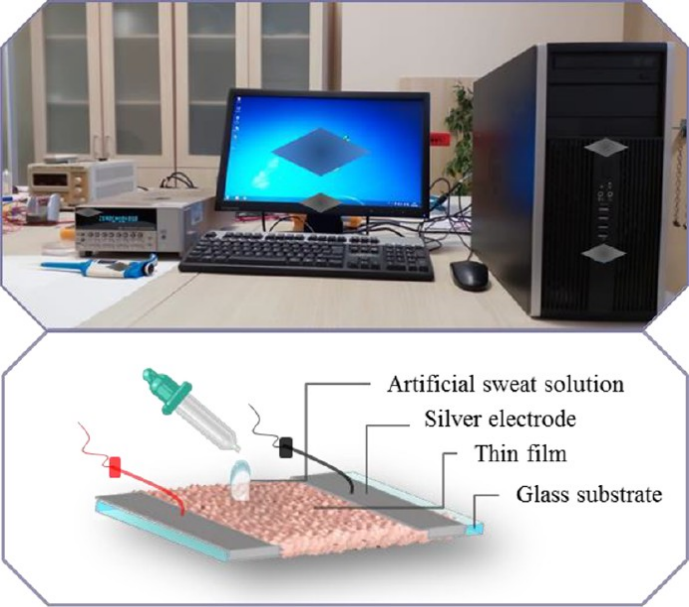
Photograph experimental setup of conductometric continuous sweat loss tracking sensor based on nanostructured CuO thin films functionalized with *Lawsonia inermis* leaf extract.

Sweat-loss ratio tracking manners of the fabricated devices were examined by applying five different concentrations of artificial sweat solution droplets on thin-film materials privately and assessing the conductivity between the two electrodes (Figure 6). The conductivity variation was examined before and after the sweat solution droplet implementation. Real-time sweat loss sensing responses (*S*) of the fabricated CuO-based samples were calculated because of the alteration in conductance:^69^
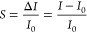
2where *I* and *I*_0_ are the obtained currents with and without artificial sweat implementations, respectively.

**Figure 7:**
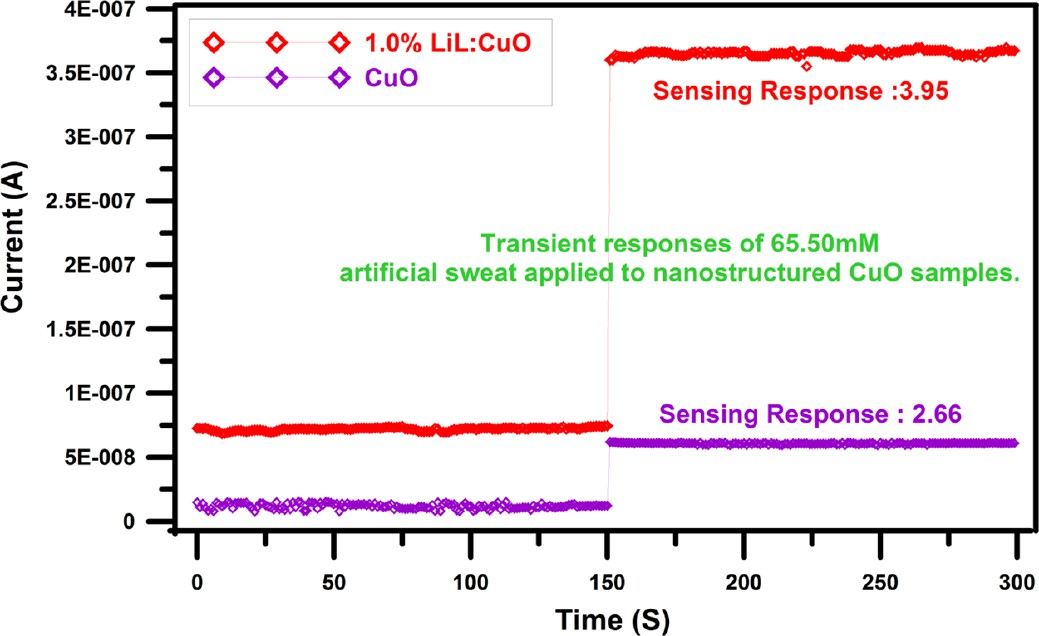
Transient response of the pristine and 1.0% LiL-substituted devices to the sweat solution (65.50 mM) droplets. It is apparent that the sensing response quality severely improves with 1.0% LiL substituting in the CuO nanostructure. It can be clearly seen, when artificial sweat is performed, the conductance value of the device is increased and then the steady state is acquired.

Figure 7 shows the transient response of 65.50 mM artificial sweat ingredients used in pristine and 1.0% LiL:CuO devices, respectively. It is clear that the sensing response quality severely improves with 1.0% LiL substituting in the CuO nanostructure. It can be seen when artificial sweat is performed, the conductance value of the device is increased and then the steady state is acquired. Despite the pristine film being responsive to the 65.50 mM concentrations (*S* = 2.66), the response characteristic improved to 3.95 for the 1.0% LiL-implemented CuO film. Both samples exhibit remarkable consistency with and without droplet usage. Because of the hydroxyl group, henna presents a great artificial sweat solution absorption performance.

Also, Figure 7 presents that the conductivity value of the unmodified CuO sample is enhanced as a result of LiL implementation, which signs a reduction in a specific contact resistance of the thin-film structure. This reduction in resistance could be attributed to the adjustment of some physical parameters of the produced samples like crystallite size, particle distribution, and surface roughness with LiL implementation.^70^ Additionally, nanostructured metal-oxide semiconductor thin-film materials have a high surface-to-volume ratio leading to more absorption of the drop solution that reasons a significant alteration in sensing characteristics.

**Figure 8:**
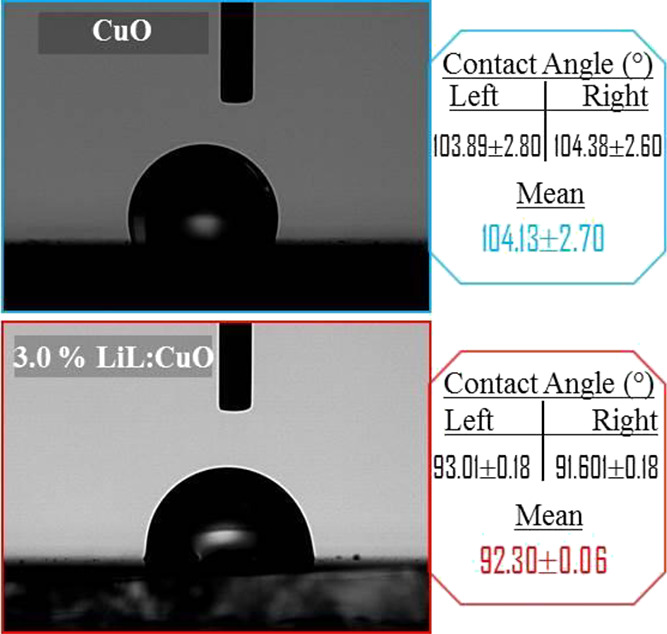
Photographs of the water contact angle CuO and 3.0% LiL:CuO synthesized with *Lawsonia inermis* leaf extract. Measured water contact angle values of thin films (right–left side and mean contact angle).

Figure 8 shows the top views of artificial sweat drops on CuO and 3.0% LiL:CuO sample surfaces, respectively. The contact angle for the pristine CuO sample surface was found to be 103.89°. However, after the usage of LiL, the surface was found to be hydrophilic with a contact angle of 91.68°. It can be seen that LiL added in the growth bath can enhance the hydrophilicity of the surface considerably. Hence, this increases the artificial sweat interaction with the sample surface. This interaction between the solution and the fabricated sample surface is powerful for a device and leading an important sensing response. Absorbing more electrolytes in the device structure is critical to boosting the active sites on the sample surface and advances sensing abilities.^71,72^ By the way, it was reported that smooth surfaces decrease the contact angle of liquid implementation, increasing the sensing efficacy.^73^ Hence, less rough surfaces would result in preferable sensing abilities in terms of viability and repeatability. Obtained AFM images of fabricated films, revealing the reduction of SR parameters with LiL substituting (Table 1).

**Figure 9:**
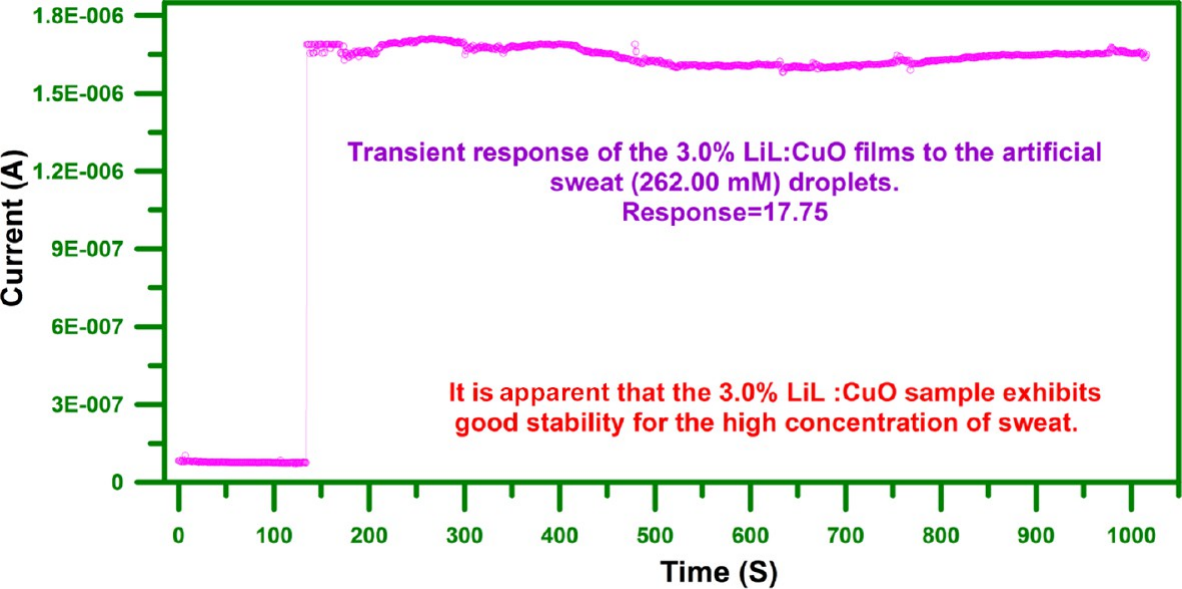
Transient response of the 3.0% LiL-substituted device to the sweat solution (262.00 mM) droplets. It becomes evident that 3.0% LiL-substituted CuO sample exhibits good stability for the high concentration of artificial sweat implementation.

When the real-time monitoring process, the response characteristics not only mean high response time but also suppose long-term stability. In accordance with this, a long-term stability survey was recorded on the 3.0% LiL-substituted device toward 262.00 mM of artificial sweat solutions for an entire period of 850 s, as illustrated in Figure 9. It becomes evident that the 3.0% LiL-substituted CuO sample exhibits good stability for the high concentration of sweat implementation.

These sensors’ limit of detection (LOD) values were also estimated using the calibration graph (concentration of artificial sweat versus current).^74^ Unmodified CuO was found to exhibit a low value of the LOD (3.60 mM) additionally, and a conspicuous reduction in the LOD was obtained with a change of LiL concentrations in the growth bath 3.10 and 2.85 mM for 1.0 and 3.0% LiL:CuO samples, respectively.

Further, the sweat response examinations were conducted for a partially long period, namely for ≈2 months (8 weeks) at room temperatures, to observe long-term reliability and the reusability of fabricated devices. The real-time sweat loss sensing response was reduced by about 6.0% after 8 weeks. The cause for this weak decrement in response after 2 months is principally owing to the satiety of the device structure for adsorption of implemented sweat solution ions such as potassium, sodium, and chloride. Since the second implementation of the same sweat solution on the device could be leading to poor interactions between the sweat ions and the device structure.

**Figure 10:**
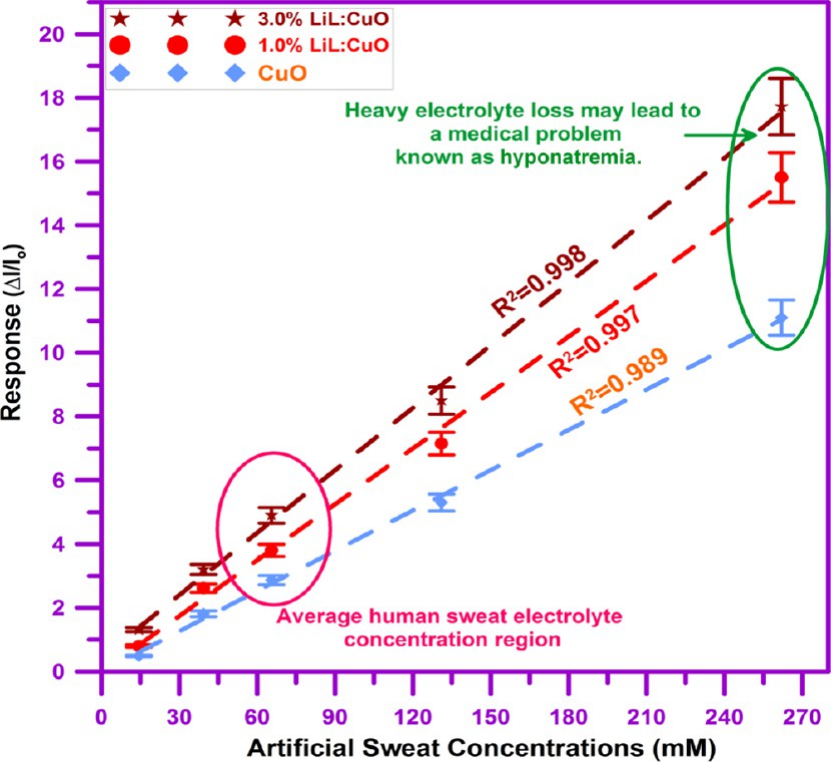
Sweat solution-sensing response for unsubstituted, 1.0 and 3.0% LiL-substituted nanostructured CuO thin-film-based devices for sweat ingredients from 14.41 to 262.00 mM. It is conspicuous that LiL concentration increase in the growth bath the sensing-response values of the fabricated devices notably, which is over definite for higher components of sweat solution.

Figure 10 illustrates the sweat solution-sensing response for unsubstituted, 1.0 and 3.0% LiL-implemented nanostructured CuO thin-film-based devices for sweat solutions from 14.41 to 262.00 mM. All fabricated devices were examined with five diverse components of artificial sweat, and the sensing-response rates are exhibited in Figure 10. These five diverse components of artificial sweat were chosen to include high and low concentrations of sweat concerning widespread content, which is approximately 70 mM. It is conspicuous that the LiL concentration increased in the growth bath the sensing-response values of the fabricated devices notably, which is over definite for higher components of sweat solution.

Figure 10 further shows the linear fit of the fabricated device sensing performances because of the sweat solution concentration ratio. It is discernible that both pristine and functionalized devices demonstrate a linear response for the five different concepts of solution. Unmodified, 1.0% LiL- and 3.0% LiL-substituted thin-film materials assure considerable linearity with linear regression ranges, *R*^2^, of 0.989, 0.997, and 0.998, respectively.

This low-cost and repeatable nanostructured CuO thin-film-based real-time sweat loss tracking methodology will help us further investigate thin-film-based new sensing equipment. Unmodified and modified CuO samples present nanostructure constitutions as discussed before. Hence, their much greater surface area per unit volume ratio and surface-free energy made them has a significant candidate for sensing operations.

The noteworthy improvement in sweat-sensing response with the LiL addition in the CuO structure is presumably a result of the change in electrochemical behavior at the sample structure because of the charge-transfer process in the systems.^68^ The studies on the electrochemical behavior of quinone and its derivatives in aqueous or aprotic solutions are critical, especially for the explanation of the charge-transfer process in biological systems. Although there are different explanations for the reduction mechanism, such as, depending on the pH value of the solution, two protons two electrons, or two electrons only.^68,75^ Besides, intermolecular charge transfer was observed between the metal and the quinone radical, and this interaction stabilizes the radical species formed upon reduction of the quinone, in the presence of a metal ion.^68,76^

**Figure 11:**
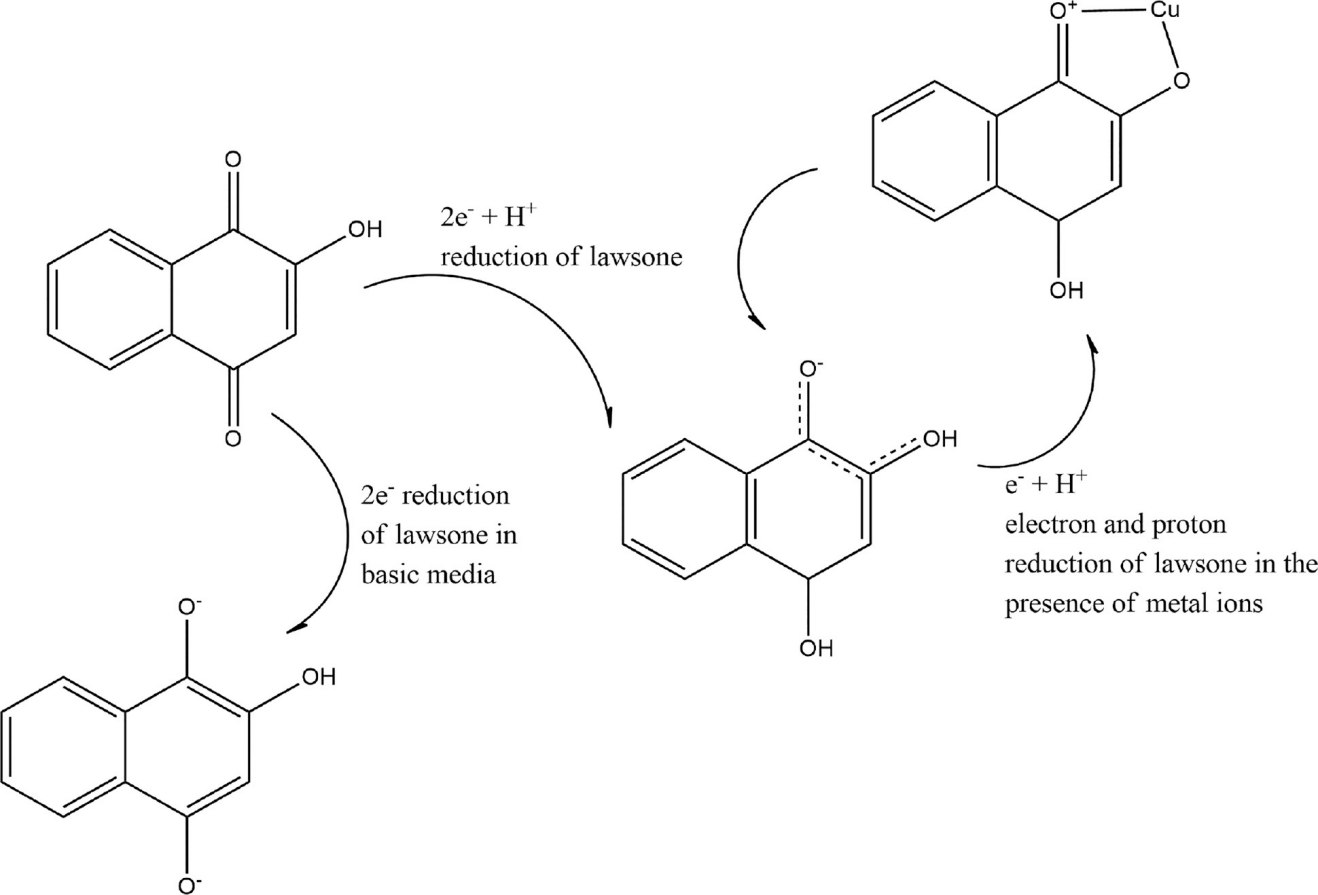
Possible two-electron reduction (in basic media) and two-electron one proton reduction mechanism in lawsone molecule (in aqueous buffer). Radicals stabilized with the Cu ions.

Lawsone is a natural *para*-quinone and additionally, it has a hydroxyl group in the vicinity of the quinone group. So, the deprotonation of this group creates a certain *ortho*-quinone radical, which this anion presents resonant structures (see Figure 11).^76^ This molecule tends to redox reactions involving both electron and proton transfer, and anions are stabilized by hydrogen bonding involving solvent water molecules even in aprotic media.^68,76^ Therefore, this deprotonation process and resonant structure are possibly sensitive to ions in sweat because these ions could contribute to the stabilization of molecules and the sensing response of henna.

Based on this outcome, it is readily clear that fabricated CuO-based sensing systems responded to sweat solutions with good response, which is reasonably encouraging for the LiL-modified CuO film structure to be used as a sweat loss-tracking material.

## 3 Conclusions

In this study, the morphological, structural, electrical, and wettability properties of CuO films with LiL (1.0 and 3.0%) in different contents and without LiL prepared using the SILAR method and sweat detection performance in sensor application were investigated. From the FESEM and AFM images, it is seen that the surface shape of the films showed significant differences with the inclusion of La, that is, it changed from a dandelion-like structure to an elliptical-like structure. All films produced according to XRD data exhibit a monoclinic lattice structure and show a decrease in crystallite size from 12.44 to 10.59 nm according to the increasing LiL percentage in the reaction bath. FT-IR analysis confirmed CuO’s stretched metal oxide vibration bond. From the wettability results, it is understood that the CuO film with 3.0% LiL shows better hydrophilic properties with a smaller water contact angle (approximately 92.30). Compared to the CuO film, the 3.0% LiL:CuO film appears to have a better sweat detection response. Therefore, the LiL-modified CuO film structure may be useful in real-time sweat-loss monitoring applications for personalized health tracking.

In the last decade, extensive investigations have been achieved in providing real-time tracking of sweat loss. Various sensing materials will need to be integrated to gain a comprehensive picture of health status. This fabricated nanostructured CuO-based sensing equipment will ensure beneficial and dependable tracking of hydration status before, during, and after exercise. As developed equipment maintains to influence sensing materials, we hope our enhancing the real-time sweat loss tracking capabilities of nanostructured CuO materials will ensemble ensure different selections to fabricate highly sensitive equipment.

## 4 Experimental

### 4.1 The Synthesis of CuO Films as a Function of LiL Percentage in the Synthesis Solutions of the SILAR Procedure

All chemicals supplied by Merck and Sigma-Aldrich Company and used in the synthesis process in the experiment are of analytical quality. Cupric chloride dihydrate (Cl_2_CuH_4_O_2_, ≥99.0%) and *L. inermis* leaf extract were used as Cu^2+^ ion resource, capping, and reducing agents, respectively. Also, in all experiments, sulfuric acid (H_2_SO_4_, 98.0%), acetone (CH_3_COCH_3_, ≥99.5%), and pure water were used to clean glass slides and beakers.

In this experiment, CuO films with and without LiL were obtained on glass slides by the SILAR way at normal pressure. For the preparation of CuO films, 0.1 M Cl_2_CuH_4_O_2_ melted in 100 mL of pure water was used as Cu^2+^ ion. The pH of the solution was adjusted to 10 by adding ammonium hydroxide (NH_4_OH). Then, the temperature of the pure water and Cu solution was kept constant at about 85°. A characteristic SILAR cycle can be defined as follows: First, the glass substrate was immersed in the solution for 20 s to adsorb the Cu^2+^ ions on the glass. The substrate was then immersed in distilled water to remove weakly bound Cu^2+^ ions from the glass. This procedure was repeated 12 times to deposit smooth and homogeneous CuO films. To investigate the effect of LiL extract on CuO films, LiL was added to the reaction solution as a reducing agent. Two series of CuO films with 1.0 and 3.0% LiL was obtained. Finally, all the synthesized CuO films were annealed in an oven at 250 °C for 45 min.

### 4.2 Materials’ Characterizations

The fabricated CuO samples were characterized by field emission scanning electron microscopy (FESEM), model Zeiss Supra 55 fitted with an energy-dispersive X-ray spectrometry (EDX) equipment for conducting elemental composition and X-ray Diffraction (XRD), model Bruker D8 advance. The thicknesses of the produced thin films were measured using a NanoMap 500LS 3D profilometer. The surface topography of the films was operated by Solaris AFM. FT-IR measurements were conducted to examine the functional group or chemical bonding of samples in the spectral region 400–4000 cm^–1^ using a Shimadzu IRAffinity-1S. The electrical characterization current–time (*I*–*t*) was investigated using a Keithley 6487 source meter under room conditions.
